# Supplementary material for: Thymocytes trigger self-antigen-controlling pathways in immature medullary thymic epithelial stages
Source: eLife. 2022 Feb 21;11:e69982. doi: 10.7554/eLife.69982 (PMC8860447; doi:10.7554/eLife.69982)
Supplement: Supplementary file 1. [file elife-69982-supp1.pdf]

Supplementary file 1

AIRE_dependent_FEZF2_independent TRAs		
**Gene ID**	**Gene symbol**	**mRNA Accession number**
69364	1700012P22Rik	NM_027056.1
70882	Armc3	NM_001081083.2
11435	Chrna1	NM_007389.5
26887	Chst4	NM_011998.4
76703	Cpb1	NM_029706.2
240058	Cpne5	NM_001360239.1
12918	Crh	NM_205769.3
26898	Ctsj	NM_001356291.1
13076	Cyp1a1	NM_001136059.2
75465	Dynlrb2	NM_029297.1
52614	Emr4	NM_139138.3
77767	Ermn	NM_029972.3
240121	Fsd1	NM_183178.2
14371	Fzd9	NM_010246.1
14531	Gcm1	NM_008103.3
14621	Gjb4	NM_008127.4
15162	Hck	NM_001172117.1
16069	Igj	NM_152839.3
69540	Klk10	NM_133712.2
74354	Lrguk	NM_028886.1
76668	Mdh1b	NM_029696.4
104362	Meig1	NM_001355205.1
18039	Nefl	NM_010910.2
18133	Nov	NM_010930.4
64011	Nrgn	NM_022029.2
18976	Pomc	NM_001278581.1
18776	Prl3b1	NM_008865.3
72373	Psca	NM_028216.2
72242	Psg21	NM_027403.4
380683	Sec14l3	NM_001029937.2
20604	Sst	NM_009215.1
70956	Tex19.2	NM_027622.3
381107	Tmem232	NM_001008973.2
76670	Ttc18	NM_001163638.1
73301	Ttc29	NM_183096.3
69480	Ttc9	NM_001033149.3
72094	Ugt2a3	NM_028094.3
71860	Wdr16	NM_027963.2
22403	Wisp2	NM_016873.2
22773	Zic3	NM_009575.2

FEZF2_dependent_AIRE_independent TRAs		
**Gene ID**	**Gene symbol**	**mRNA Accession number**
14128	Fcer2a	NM_001253737.1
15002	H2-Ob	NM_010389.3
16521	Kcnj5	NM_010605.5
246313	Prokr2	NM_144944.3
246709	Rgs13	NM_153171.4

AIRE_dependent_FEZF2_dependent TRAs		
**Gene ID**	**Gene symbol**	**mRNA Accession number**
16678	Krt1	NM_008473.2
19692	Reg1	NM_009042.2

AIRE_independent_FEZF2_independent TRAs		
**Gene ID**	**Gene symbol**	**mRNA Accession number**
75556	1700026D08Rik	NM_029335.3
239559	A4galt	NM_001004150.3
110751	Adam33	NM_001163529.2
108153	Adamts7	NM_001003911.2
224129	Adcy5	NM_001012765.5
11689	Alox5	NM_009662.2
64933	Ap3m2	NM_001122820.1
545260	Arsi	NM_001038499.1
319767	Atp10b	NM_176999.3
68127	B230217C12Rik	NM_001080935.
12012	Baat	NM_007519.3
212998	BC016579	NM_145389.2
332713	BC051628	NM_199312.3
12169	Bmx	NM_009759.4
547431	Btnl2	NM_079835.2
71764	C2cd2l	NM_001359259.1
245555	C77370	NM_001077354.2
260299	Cadm4	NM_153112.3
71213	Cage1	NM_027724.2
12354	Car7	NM_001301164.1
228942	Cbln4	NM_175631.3
207607	Ccdc40	NM_175430.4
70445	Cd248	NM_054042.2
102657	Cd276	NM_133983.4
12647	Chat	NM_009891.2
108699	Chn1	NM_001113246.2
83453	Chrdl1	NM_001114385.1
12265	Ciita	NM_001243760.2
56863	Cldn9	NM_020293.3
12944	Crp	NM_007768.4
320127	Dgki	NM_001081206.2
13449	Dok2	NM_010071.2

14073	Faah	NM_010173.5
14238	Foxf2	NM_010225.2
15223	Foxj1	NM_008240.3
64337	Gng13	NM_001357782.1
14998	H2-DMa	NM_001360530.1
14999	H2-DMb1	NM_010387.3
15000	H2-DMb2	NM_010388.4
381091	H2-Eb2	NM_001033978.3
15001	H2-Oa	NM_008206.2
66438	Hamp2	NM_183257.4
15114	Hap1	NM_001359052.1
171283	Havcr1	NM_001166631.1
16153	Il10	NM_010548.2
50905	Il17rb	NM_019583.3
16408	Itgal	NM_001253872.1
16420	Itgb6	NM_001159564.1
16891	Lipg	NM_010720.3
16970	Lrmp	NM_001281980.1
16977	Lrrc23	NM_001302555.1
17179	Matk	NM_001285853.1
50913	Olig2	NM_016967.2
214424	Parp16	NM_177460.4
279653	Pcdh19	NM_001105245.1
320207	Pik3r5	NM_177320.2
104709	Pik3r6	NM_001004435.3
241113	Prkag3	NM_153744.3
246710	Rhobtb2	NM_153514.5
67071	Rps6ka6	NM_025949.3
22092	Rsph1	NM_025290.3
56788	Scube2	NM_020052.2
74478	Snx29	NM_001290148.1
223227	Sox21	NM_177753.3
20680	Sox7	NM_011446.1
68792	Srpx2	NM_001083895.3
20849	Stat4	NM_001308266.1
21687	Tek	NM_001290549.1
381339	Tmem182	NM_001081198.1
140765	Tmprss3	NM_001163776.1
210801	Unc5d	NM_001347498.1
100647	Upk3b	NM_175309.4
245595	Zfp711	NM_177747.3
