# Supplementary material for: Thymocytes trigger self-antigen-controlling pathways in immature medullary thymic epithelial stages
Source: eLife. 2022 Feb 21;11:e69982. doi: 10.7554/eLife.69982 (PMC8860447; doi:10.7554/eLife.69982)
Supplement: Supplementary file 2. [file elife-69982-supp2.pdf]

Supplementary file 2

AIRE_dependent_FEZF2_independent TRAs		
**Gene ID**	**Gene symbol**	**mRNA Accession number**
69318	1700007K09Rik	NM_027037.2
69325	1700012B09Rik	NM_029306.3
73634	1700125H20Rik	NM_028589.1
69457	2310005G13Rik	NM_001358101.1
381816	4922502D21Rik	NM_199034.3
11451	Acrv1	NM_007391.2
105349	Akr1c18	NM_001346535.1
76768	Alpi	NM_001081082.2
11647	Alpl	NM_001287172.1
11694	Alx3	NM_007441.3
71724	Aox3	NM_023617.2
11807	Apoa2	NM_001305549.1
244867	Arhgap20	NM_175535.3
382000	AY761184	NM_001007582.3
81799	C1qtnf3	NM_001204134.1
234964	Ccdc67	NM_181816.2
20299	Ccl22	NM_009137.2
218630	Ccno	NM_001081062.1
170786	Cd209a	NM_133238.5
170779	Cd209d	NM_130904.2
69142	Cd209f	NM_026956.2
12502	Cd3g	NM_009850.2
66996	Ceacam11	NM_023289.2
17474	Clec4d	NM_001163161.1
51811	Clec4f	NM_016751.3
76703	Cpb1	NM_029706.2
12891	Cpne6	NM_001136057.3
12903	Crabp1	NM_001284507.1
12918	Crh	NM_205769.3
12954	Cryaa	NM_001278569.1
105859	Csdc2	NM_145473.3
26898	Ctsj	NM_001356291.1
55985	Cxcl13	NM_018866.2
110115	Cyp11b1	NM_001033229.3
13077	Cyp1a2	NM_009993.3
382059	Defa22	NM_207658.4
13386	Dlk1	NM_001190703.1
21673	Dntt	NM_001043228.1
75465	Dynlrb2	NM_029297.
105418	E330034G19Rik	NM_001033214.2
77767	Ermn	NM_029972.3
16204	Fabp6	NM_008375.2
14347	Fut7	NM_001177366.1
14531	Gcm1	NM_008103.3

76743	Gje1	NM_029722.1
14780	Gpx5	NM_010343.2
75512	Gpx6	NM_145451.3
68312	Gstm7	NM_001356351.1
232493	Gys2	NM_145572.2
14939	Gzmb	NM_013542.3
15492	Hsd3b1	NM_001304800.1
16069	Igj	NM_152839.3
16334	Ins2	NM_001185083.2
233752	Insc	NM_173767.3
16399	Itga2b	NM_010575.2
17992	Itih3	NM_008407.2
16533	Kcnmb1	NM_031169.4
13648	Klk1b9	NM_010116.1
268482	Krt12	NM_010661.2
16682	Krt4	NM_008475.2
227630	Lcn11	NM_001100455.2
16858	Lgals7	NM_008496.4
17195	Mbl2	NM_010776.1
17844	Mup5	NM_008649.2
17897	Myl3	NM_001364484.1
17926	Myoc	NM_010865.3
67405	Nts	NM_024435.2
18379	Omt2a	NM_001111286.2
242726	Padi6	NM_153106.2
10982	Pgc	NM_025973.3
18716	Pip	NM_008843.4
57749	Piwil1	NM_021311.3
69836	Pla2g12b	NM_023530.2
18778	Pla2g1b	NM_001356586.1
18976	Pomc	NM_001278581.1
18776	Prl3b1	NM_008865.3
72373	Psca	NM_028216.2
217212	Pyy	NM_001346771.1
109222	Rarres1	NM_001164763.1
63954	Rbp7	NM_022020.2
19674	Rcvrn	NM_009038.2
20284	Scrg1	NM_009136.
20706	Serpinb9b	NM_011452.2
93806	Serpinb9g	NM_011455.
20389	Sftpc	NM_011359.2
382113	Slc22a14	NM_001037749.2
209837	Slc38a5	NM_172479.3
20763	Sprr2i	NM_011475.3
20765	Sprr2k	NM_011477.3
21334	Tac2	NM_001199971.1
21823	Th	NM_009377.2
22074	Try4	NM_011646.5

76670	Ttc18	NM_001163638.1
72094	Ugt2a3	NM_028094.3
242122	Vtcn1	NM_178594.3
71860	Wdr16	NM_027963.2
114602	Zmynd10	NM_001364526.1

FEZF2_dependent_AIRE_independent TRAs		
**Gene ID**	**Gene symbol**	**mRNA Accession number**
108956	Apol7c	NM_175391.4
12307	Calb1	NM_009788.4
12810	Coch	NM_001198835.1
12140	Fabp7	NM_021272.3
15002	H2-Ob	NM_010389.3
68678	Smtnl1	NM_024230.2
69083	Sult1c2	NM_026935.4

AIRE_dependent_FEZF2_dependent TRAs		
**Gene ID**	**Gene symbol**	**mRNA Accession number**
11806	Apoa1	NM_009692.4
11808	Apoa4	NM_007468.2
11813	Apoc2	NM_001277944.1
12308	Calb2	NM_007586.1
73708	Dppa3	NM_139218.1
21884	Fabp9	NM_011598.3
14864	Gstm3	NM_010359.2
18478	Pah	NM_008777.3
19692	Reg1	NM_009042.2
20209	Saa2	NM_001357491.1

AIRE_independent_FEZF2_independent TRAs		
**Gene ID**	**Gene symbol**	**mRNA Accession number**
74230	1700016K19Rik	NM_198637.2
69528	1700030J22Rik	NM_001357253.1
235973	A630095E13Rik	NM_001033325.2
18670	Abcb4	NM_008830.2
100705	Acacb	NM_133904.2
11489	Adam12	NM_007400.2
110751	Adam33	NM_001163529.2
11516	Adcyap1	NM_001315503.1
329738	Aknad1	NM_177859.3
11812	Apoc1	NM_001110009.2
11945	Atp4b	NM_009724.2
20877	Aurkb	NM_011496.2
68127	B230217C12Rik	NM_001080935.1
27062	Cadps	NM_001042617.1
12310	Calca	NM_001033954.3
116903	Calcb	NM_054084.2
20296	Ccl2	NM_011333.3

20304	Ccl5	NM_013653.3
12500	Cd3d	NM_013487.3
12501	Cd3e	NM_007648.5
12525	Cd8a	NM_001081110.2
12526	Cd8b1	NM_009858.3
107934	Celsr3	NM_001359572.1
12649	Chek1	NM_007691.5
12265	Ciita	NM_001243760.2
57255	Cldn13	NM_020504.4
20256	Clec11a	NM_009131.3
12919	Crhbp	NM_198408.3
11571	Crisp1	NM_009638.3
12960	Crybb1	NM_001312893.1
12991	Csn2	NM_001286020.1
12477	Ctla4	NM_001281976.1
13048	Cux2	NM_001312908.1
13363	Dhh	NM_007857.5
239133	Dleu7	NM_173419.2
252864	Dusp15	NM_001159376.1
242705	E2f2	NM_001305399.1
52679	E2f7	NM_001358560.1
13607	Eda	NM_001177937.1
69306	Efcab9	NM_027031.3
71877	Efhc1	NM_027974.1
215900	Fam26f	NM_175449.4
56636	Fgf21	NM_020013.4
14255	Flt3	NM_010229.2
242022	Frem2	NM_172862.3
11936	Fxyd2	NM_007503.3
14711	Gnmt	NM_010321.1
54672	Gpr97	NM_173036.3
14813	Grin2c	NM_010350.2
14998	H2-DMa	NM_001360530.1
14999	H2-DMb1	NM_010387.3
15000	H2-DMb2	NM_010388.4
381091	H2-Eb2	NM_001033978.3
15001	H2-Oa	NM_008206.2
84506	Hamp	NM_032541.2
330723	Htra4	NM_001081187.3
74096	Hvcn1	NM_001042489.2
16154	Il10ra	NM_001324486.1
16159	Il12a	NM_001159424.2
50929	Il22	NM_016971.2
404710	Iqgap3	NM_001033484.1
16425	Itih2	NM_010582.3
16551	Kif11	NM_010615.1
109242	Kif24	NM_024241.2
70810	Krt25	NM_133730.1

16818	Lck	NM_001162432.1
568988	Lrrc23	NM_001302555.1
114332	Lyve1	NM_053247.4
17123	Madcam1	NM_001358785.1
17349	Mlf1	NM_001039543.2
17427	Mns1	NM_008613.3
228785	Mylk2	NM_001081044.2
213435	Mylk3	NM_001297612.1
18159	Nppc	NM_010933.5
18197	Nsg2	NM_001290680.1
236690	Nyx	NM_173415.4
67968	Ooep	NM_026480.3
78826	P2ry10	NM_001357810.1
18610	Pdyn	NM_001286502.1
18613	Pecam1	NM_001032378.2
57757	Pglyrp2	NM_001271476.1
104709	Pik3r6	NM_001004435.3
665270	Plb1	NM_001081407.1
19074	Prg2	NM_008920.4
53856	Prg3	NM_016914.2
19132	Prph	NM_001163588.1
229214	Qrfpr	NM_198192.2
19373	Rag1	NM_009019.2
12399	Runx3	NM_019732.2
20192	Ryr3	NM_001319156.1
66166	S100a14	NM_001163525.2
56788	Scube2	NM_020052.2
68054	Serpina12	NM_026535.2
71869	Serpinb12	NM_001199213.2
105355	Slc17a3	NM_001164743.1
66166	Slc2a6	NM_001163525.2
328059	Slc7a15	NM_001038660.2
20618	Sncg	NM_011430.3
74478	Snx29	NM_001290148.1
66042	Sostdc1	NM_025312.3
66722	Spag16	NM_001271533.1
20728	Spic	NM_011461.3
100689	Spon2	NM_133903.3
20257	Stmn2	NM_025285.2
20897	Stra6	NM_001162475.1
57765	Tbx21	NM_019507.2
21414	Tcf7	NM_001313981.1
217733	Tmem63c	NM_001361704.1
228775	Trib3	NM_175093.2
239364	Tspyl5	NM_001085421.1
22160	Twist1	NM_011658.2
328795	Ubash3a	NM_177823.4
24108	Ubd	NM_023137.3

109637	Upk1a	NM_026815.2
100647	Upk3b	NM_175309.4
