# Supplementary material for: Thymocytes trigger self-antigen-controlling pathways in immature medullary thymic epithelial stages
Source: eLife. 2022 Feb 21;11:e69982. doi: 10.7554/eLife.69982 (PMC8860447; doi:10.7554/eLife.69982)
Supplement: Supplementary file 3. [file elife-69982-supp3.pdf]

**Supplementary file 3**

| AIRE_dependent_FEZF2_independent TRAs | | |
|---|---|---|
| **Gene ID** | **Gene symbol** | **mRNA Accession number** |
| 67082 | 1700011H14Rik | NM_025956.4 |
| 66732 | 4921530L21Rik | NM_001358241.1 |
| 319942 | A530016L24Rik | NM_177039.4 |
| 239435 | Aard | NM_175503.3 |
| 219033 | Ang4 | NM_177544.4 |
| 11998 | Avp | NM_009732.2 |
| 212989 | Best2 | NM_001130194.1 |
| 12163 | Bmp8a | NM_001256019.1 |
| 29866 | Cabp2 | NM_001160253.1 |
| 20297 | Ccl20 | NM_001159738.1 |
| 20307 | Ccl8 | NM_021443.3 |
| 386463 | Cdsn | NM_001008424.2 |
| 71601 | Ceacam20 | NM_027839.2 |
| 109791 | Clps | NM_001317065.1 |
| 12903 | Crabp1 | NM_001284507.1 |
| 208677 | Creb3l3 | NM_145365.3 |
| 13012 | Cst8 | NM_009978.2 |
| 55985 | Cxcl13 | NM_018866.2 |
| 13070 | Cyp11a1 | NM_001346787.1 |
| 13075 | Cyp19a1 | NM_001348171.1 |
| 13076 | Cyp1a1 | NM_001136059.2 |
| 13077 | Cyp1a2 | NM_009993.3 |
| 13087 | Cyp2a5 | NM_007812.4 |
| 68171 | D730048I06Rik | NM_026593.3 |
| 13206 | Ddx4 | NM_001145885.1 |
| 50722 | Dkkl1 | NM_015789.3 |
| 13386 | Dlk1 | NM_001190703.1 |
| 15572 | Elavl4 | NM_001038698.1 |
| 207393 | Elfn2 | NM_001358692.1 |
| 270162 | Elmod1 | NM_177769.4 |
| 75429 | Fam183b | NM_001162878.2 |
| 229499 | Fcrl1 | NM_001136236.1 |
| 14170 | Fgf15 | NM_008003.2 |
| 14526 | Gcg | NM_008100.4 |
| 14607 | Gip | NM_008119.2 |
| 66283 | Gkn1 | NM_025466.1 |
| 66284 | Gkn2 | NM_025467.1 |
| 14765 | Gpr50 | NM_001308501.1 |
| 14916 | Guca2b | NM_008191.2 |
| 14939 | Gzmb | NM_013542.3 |
| 15162 | Hck | NM_001172117.1 |
| 72927 | Hepacam | NM_175189.4 |
| 101202 | Hepacam2 | NM_178899.5 |
| 15408 | Hoxb13 | NM_008267.4 |

| | | |
|---|---|---|
| 15492 | Hsd3b1 | NM_001304800.1 |
| 72413 | Kcnmb2 | NM_028231.2 |
| 19144 | Klk6 | NM_001164696.1 |
| 23993 | Klk7 | NM_011872.3 |
| 16644 | Kng1 | NM_001102411.1 |
| 433619 | Kprp | NM_028629.1 |
| 406220 | Krt77 | NM_006521140.1 |
| 332131 | Krt78 | NM_212487.4 |
| 328789 | Lhfpl5 | NM_026571.2 |
| 17182 | Matn3 | NM_010770.4 |
| 50997 | Mpp2 | NM_001356324.1 |
| 14126 | Ms4a2 | NM_001276328.1 |
| 17771 | Mtl5 | NM_001039657.2 |
| 18133 | Nov | NM_010930.4 |
| 23958 | Nr2e3 | NM_013708.4 |
| 67405 | Nts | NM_024435.2 |
| 100535 | Oas1d | NM_133893.3 |
| 21906 | Otop1 | NM_172709.3 |
| 109820 | Pgc | NM_025973.3 |
| 53906 | Phgr1 | NM_001145644.1 |
| 18976 | Pomc | NM_001278581.1 |
| 22264 | Prap1 | NM_009475.2 |
| 69814 | Prss32 | NM_027220.2 |
| 19259 | Ptpn5 | NM_001163565.1 |
| 19662 | Rbp4 | NM_001159487.1 |
| 19694 | Reg3a | NM_011259.1 |
| 18489 | Reg3b | NM_011036.1 |
| 19695 | Reg3g | NM_011260.2 |
| 67709 | Reg4 | NM_026328.2 |
| 67792 | Rgs8 | NM_001347115.1 |
| 19752 | Rnase1 | NM_011271.2 |
| 104582 | Rprml | NM_001033212.2 |
| 380683 | Sec14l3 | NM_001029937.2 |
| 20706 | Serpinb9b | NM_011452.2 |
| 210027 | Slc35f3 | NM_001363500.1 |
| 209837 | Slc38a5 | NM_172479.3 |
| 28250 | Slco1a4 | NM_001355577.1 |
| 57277 | Slurp1 | NM_020519.1 |
| 20754 | Sprr1b | NM_009265.3 |
| 20604 | Sst | NM_009215.1 |
| 56362 | Sult1b1 | NM_001356943.1 |
| 72003 | Synpr | NM_001163032.1 |
| 21334 | Tac2 | NM_001199971.1 |
| 21338 | Tacr3 | NM_021382.6 |
| 21786 | Tff3 | NM_011575.2 |
| 208613 | Tmem212 | NM_001164437.1 |
| 50930 | Tnfsf14 | NM_019418.3 |
| 329278 | Tnn | NM_177839.3 |

| | | |
|---|---|---|
| 195359 | Trim40 | NM_001033235.3 |
| 76670 | Ttc18 | NM_001163638.1 |
| 242800 | Ttc34 | NM_172878.3 |
| 192653 | Ttc36 | NM_138951.1 |

| FEZF2_dependent_AIRE_independent TRAs | | |
|---|---|---|
| **Gene ID** | **Gene symbol** | **mRNA Accession number** |
| 108956 | Apol7c | NM_175391.4 |
| 68891 | Cd177 | NM_026862.3 |
| 12565 | Cdh9 | NM_009869.1 |
| 12140 | Fabp7 | NM_021272.3 |
| 54713 | Fezf2 | NM_080433.3 |
| 71520 | Grap | NM_027817.3 |
| 72585 | Lypd1 | NM_001311089.1 |
| 17287 | Mep1a | NM_008585.2 |
| 319211 | Nol4 | NM_001161483.1 |
| 246313 | Prokr2 | NM_144944.3 |
| 19711 | Resp18 | NM_009049.2 |
| 246709 | Rgs13 | NM_153171.4 |
| 72902 | Spock3 | NM_001252620.1 |
| 69083 | Sult1c2 | NM_026935.4 |
| 241556 | Tspan18 | NM_183180.2 |

| AIRE_dependent_FEZF2_dependent TRAs | | |
|---|---|---|
| **Gene ID** | **Gene symbol** | **mRNA Accession number** |
| 78354 | 2210407C18Rik | NM_144544.2 |
| 230163 | Aldob | NM_144903.3 |
| 11806 | Apoa1 | NM_009692.4 |
| 11808 | Apoa4 | NM_007468.2 |
| 11813 | Apoc2 | NM_001277944.1 |
| 12319 | Car8 | NM_007592.3 |
| 268663 | Cdhr2 | NM_001033364.3 |
| 13009 | Csrp3 | NM_001198841.1 |
| 21884 | Fabp9 | NM_011598.3 |
| 78354 | Myh3 | NM_144544.2 |
| 243262 | Oas1f | NM_145153.3 |
| 18478 | Pah | NM_008777.3 |
| 20208 | Saa1 | NM_001357493. |
| 20210 | Saa3 | NM_011315.3 |
| 66198 | Them5 | NM_025416.3 |
| 226654 | Tstd1 | NM_001164525.1 |

| AIRE_independent_FEZF2_independent TRAs | | |
|---|---|---|
| **Gene ID** | **Gene symbol** | **mRNA Accession number** |
| 71775 | 1300017J02Rik | NM_027918.2 |
| 72301 | 1810041L15Rik | NM_001163145.1 |
| 70291 | 2510049J12Rik | NM_001101431.1 |
| 69865 | A1cf | NM_001081074.1 |

| | | |
|---|---|---|
| 100705 | Acacb | NM_133904.2 |
| 11423 | Ache | NM_001290010.1 |
| 11474 | Actn3 | NM_013456.2 |
| 231821 | Adap1 | NM_006504669.2 |
| 432530 | Adcy1 | NM_009622.1 |
| 11548 | Adra1b | NM_001284380.1 |
| 11689 | Alox5 | NM_009662.2 |
| 68737 | Angel1 | NM_144524.2 |
| 71872 | Aox4 | NM_023631.2 |
| 56380 | Arid3b | NM_019689.2 |
| 17172 | Ascl1 | NM_008553.5 |
| 67855 | Asprv1 | NM_026414.2 |
| 11567 | Avil | NM_009635.3 |
| 93961 | B3galt5 | NM_001122993.1 |
| 12022 | Barx1 | NM_007526.4 |
| 12038 | Bche | NM_009738.3 |
| 12169 | Bmx | NM_009759.4 |
| 207777 | Bzrap1 | NM_172449.2 |
| 72709 | C1qtnf6 | NM_001204152.1 |
| 12287 | Cacna1b | NM_001042528.2 |
| 12292 | Cacna1s | XM_006529106.2 |
| 27062 | Cadps | NM_001042617.1 |
| 12310 | Calca | NM_001033954.3 |
| 116903 | Calcb | NM_054084.2 |
| 80796 | Calm4 | NM_020036.4 |
| 434778 | Ccdc160 | NM_001034059.1 |
| 24047 | Ccl19 | NM_011888.2 |
| 12467 | Cct6b | NM_001291242.1 |
| 21939 | Cd40 | NM_011611.2 |
| 15985 | Cd79b | NM_001313939.1 |
| 12564 | Cdh8 | NM_001039154.2 |
| 72040 | Cdhr5 | NM_001114322.1 |
| 12590 | Cdx1 | NM_009880.3 |
| 26366 | Ceacam10 | NM_007675.4 |
| 18636 | Cfp | NM_008823.4 |
| 12647 | Chat | NM_009891.2 |
| 229933 | Clca2 | XM_006501432.3 |
| 362053 | Clca4 | NM_201419.1 |
| 57255 | Cldn13 | NM_020504.4 |
| 212070 | Clrn3 | NM_178669.5 |
| 69700 | Col22a1 | NM_027174.1 |
| 11571 | Crisp1 | NM_009638.3 |
| 12944 | Crp | NM_007768.4 |
| 54698 | Crtam | NM_001281954.1 |
| 12971 | Crym | NM_016669.2 |
| 12983 | Csf2rb | NM_001358854.1 |
| 12991 | Csn2 | NM_001286020.1 |
| 73720 | Cst6 | NM_028623.5 |

| | | |
|---|---|---|
| 68588 | Cthrc1 | NM_026778.2 |
| 15945 | Cxcl10 | NM_021274.2 |
| 17329 | Cxcl9 | NM_008599.4 |
| 13074 | Cyp17a1 | NM_007809.3 |
| 13370 | Dio1 | NM_007860.4 |
| 239133 | Dleu7 | NM_173419.2 |
| 54485 | Dll4 | NM_019454.3 |
| 269109 | Dpp10 | NM_199021.3 |
| 13510 | Dsg1a | NM_010079.2 |
| 103551 | E130012A19Rik | NM_175332.3 |
| 242705 | E2f2 | NM_001305399.1 |
| 268780 | Egflam | NM_001289496.1 |
| 112407 | Egln3 | NM_028133.2 |
| 13807 | Eno2 | NM_001302642.1 |
| 71920 | Epgn | NM_053087.2 |
| 56226 | Espn | NM_019585.3 |
| 69627 | Fam89a | NM_001081120.1 |
| 80891 | Fcrls | NM_030707.3 |
| 14159 | Fes | NM_010194.2 |
| 59083 | Fetub | NM_001083904.1 |
| 73191 | Fezf1 | NM_028462.1 |
| 232237 | Fgd5 | NM_172731.3 |
| 14169 | Fgf14 | NM_010201.4 |
| 56636 | Fgf21 | NM_020013.4 |
| 15223 | Foxj1 | NM_008240.3 |
| 14344 | Fut2 | NM_001271993.1 |
| 210710 | Gab3 | NM_181584.4 |
| 228858 | Gdap1l1 | NM_144891.2 |
| 83408 | Gimap3 | NM_031247.3 |
| 14695 | Gnb3 | NM_013530.1 |
| 64337 | Gng13 | NM_001357782.1 |
| 238377 | Gpr68 | NM_001177673.1 |
| 15109 | Hal | NM_010401.4 |
| 15114 | Hap1 | NM_001359052.1 |
| 15464 | Hrc | NM_010473.2 |
| 330723 | Htra4 | NM_001081187.3 |
| 16005 | Igfals | NM_001364895.1 |
| 16159 | Il12a | NM_001159424.2 |
| 16178 | Il1r2 | NM_001360800.1 |
| 53626 | Insm1 | NM_016889.3 |
| 27056 | Irf5 | NM_001252382.1 |
| 16429 | Itln1 | NM_010584.3 |
| 57339 | Jph1 | NM_020604.2 |
| 60613 | Kcnq4 | NM_001081142.2 |
| 242721 | Klhdc7a | NM_173427.2 |
| 50928 | Klrg1 | NM_016970.1 |
| 76484 | Kndc1 | NM_177261.4 |
| 66809 | Krt20 | NM_023256.2 |

| | | |
|---|---|---|
| 75706 | Krt24 | NM_029393.2 |
| 15450 | Lipc | NM_001324472.1 |
| 76113 | Lpo | NM_080420.2 |
| 16970 | Lrmp | NM_001281980.1 |
| 268747 | Lrrc16b | NM_001024645.2 |
| 16992 | Lta | NM_010735.2 |
| 17001 | Ltc4s | NM_001313968.1 |
| 17005 | Ltk | NM_008523.2 |
| 217721 | Mfsd7c | NM_145447.2 |
| 17395 | Mmp9 | NM_013599.4 |
| 17922 | Myo7b | NM_032394.3 |
| 17928 | Myog | NM_031189.2 |
| 74513 | Neto2 | NM_001081324.2 |
| 269116 | Nfasc | NM_001160316.1 |
| 217431 | Nol10 | NM_001008421.1 |
| 18126 | Nos2 | NM_001313921.1 |
| 18231 | Nxph1 | NM_008751.5 |
| 380924 | Olfm4 | NM_001351947.1 |
| 57757 | Pglyrp2 | NM_001271476.1 |
| 320207 | Pik3r5 | NM_177320.2 |
| 85031 | Pla1a | XM_006522771.1 |
| 26971 | Pla2g2f | NM_001360875.1 |
| 434128 | Pnmal2 | NM_001099636.2 |
| 18985 | Pou2af1 | NM_011136.2 |
| 56635 | Prl2a1 | NM_019991.1 |
| 71373 | Prr16 | NM_001081224.2 |
| 19227 | Pthlh | NM_008970.4 |
| 20459 | Ptk6 | NM_001356304.1 |
| 56089 | Ramp3 | NM_019511.3 |
| 75141 | Rasd2 | NM_029182.1 |
| 320100 | Relt | NM_001358914.1 |
| 51791 | Rgs14 | NM_001360714.1 |
| 56533 | Rgs17 | NM_001161822.1 |
| 20129 | Rptn | NM_009100.2 |
| 72780 | Rspo3 | NM_028351.3 |
| 104001 | Rtn1 | NM_001007596.2 |
| 235281 | Scn3b | NM_001083917.1 |
| 70061 | Sdr9c7 | NM_027301.3 |
| 68054 | Serpina12 | NM_026535.2 |
| 20720 | Serpina7 | NM_009255.4 |
| 71869 | Serpinb12 | NM_001199213.2 |
| 11905 | Serpinc1 | NM_080844.4 |
| 20370 | Sez6 | NM_001291225.1 |
| 230613 | Skint10 | NM_177668.2 |
| 227659 | Slc2a6 | NM_001177627.1 |
| 239250 | Slitrk6 | NM_175499.4 |
| 276829 | Smtnl2 | NM_177776.3 |
| 20618 | Sncg | NM_011430.3 |

| | | |
|---|---|---|
| 223227 | Sox21 | NM_177753.3 |
| 272382 | Spib | NM_019866.1 |
| 100689 | Spon2 | NM_133903.3 |
| 68792 | Srpx2 | NM_001083895.3 |
| 240119 | St6gal2 | NM_001347403.1 |
| 20897 | Stra6 | NM_001162475.1 |
| 20979 | Syt1 | NM_001252341.1 |
| 21384 | Tbx15 | NM_009323.2 |
| 57765 | Tbx21 | NM_019507.2 |
| 210417 | Thsd7b | NM_172485.3 |
| 75604 | Tm4sf5 | NM_029360.3 |
| 18383 | Tnfrsf11b | NM_008764.3 |
| 21956 | Tnnt2 | NM_001130174.2 |
| 21957 | Tnnt3 | NM_001163664.1 |
| 28240 | Trpm2 | NM_138301.2 |
| 208634 | Tspan10 | NM_145363.2 |
| 69737 | Ttl | NM_027192.2 |
| 237858 | Tusc5 | NM_177709.3 |
| 24108 | Ubd | NM_023137.3 |
| 212190 | Ubxn10 | NM_001285928.1 |
| 226841 | Vash2 | NM_144879.2 |
| 381677 | Vgf | NM_001039385.1 |
