# Supplementary material for: Thymocytes trigger self-antigen-controlling pathways in immature medullary thymic epithelial stages
Source: eLife. 2022 Feb 21;11:e69982. doi: 10.7554/eLife.69982 (PMC8860447; doi:10.7554/eLife.69982)
Supplement: Supplementary file 4. [file elife-69982-supp4.pdf]

Supplementary file 4

	Gene Symbol	Name	Target organ	Fold change	P value
**Aire-dependent TRAs**	Acrv1	acrosomal vesicle protein 1	testis	6.0784	0.0004
	Akr1c18	aldo-keto reductase family 1. member C18	adrenal - ovary	3.1567	0.0257
	Alpi	alkaline phosphatase	intestine	12.8717	0.0154
	Alpl	alkaline phosphatase	liver-kidney-bone	3.9276	0.0305
	Alx3	aristaless-like homeobox 3	limb-CNS	10.1196	0.0146
	Aox3	aldehyde oxidase 3	liver	3.5179	0.0390
	Apoa2	apolipoprotein A-II	liver	5.5895	0.0448
	Arhgap20	Rho GTPase activating protein 20	brain	4.4964	0.0186
	AY761184	cDNA sequence AY761184provided	intestine	17.5496	0.0274
	C1qtnf3	C1q and tumor necrosis factor related protein 3	limb	3.7111	0.0129
	Ccdc67 (deup1)	deuterosome assembly protein 1	testis	17.5496	0.0274
	Ccl17	chemokine (C-C motif) ligand 17	lung	3.6806	0.0350
	Ccl22	chemokine (C-C motif) ligand 22	thymus - mammary gland	3.8790	0.0045
	Ccno	cyclin o	ovary	2.3969	0.0304
	Cd209a	Cd209a antigen	bladder-mammary gland	2.2155	0.0425
	Cd209d	Cd209d antigen	mammary gland	4.7161	0.0131
	Cd209f	Cd209f antigen	mammary gland	6.6064	0.0008
	Ceacam11	carcinoembryonic antigen-related cell adhesion molecule 11	placenta	9.3129	0.0001
	Cfap52	cilia and flagella associated protein 52	testis - lung	2.9049	0.0402
	Cfap70	cilia and flagella associated protein 70	testis - lung	2.7668	0.0281
	Clec4d	C-type lectin domain family 4. member d	liver-lung	5.5594	0.0087
	Clec4f	C-type lectin domain family 4. member f	liver	7.5307	0.0001
	Cpb1	carboxypeptidase B1	intestine - spleen	12.3771	0.0001
	Cpne6	Copine 6	brain	6.5138	0.0290
	Crabp1	cellular retinoic acid binding protein I	limb- CNS	5.2092	0.0076
	Crh	corticotropin releasing hormone	brain	7.9154	0.0318
	Cryaa	crystallin. alpha A	placenta - lung	9.5242	0.0110
	Csdc2	cold shock domain containing C2	brain	4.1945	0.0178
	Ctsj	cathepsin J	placenta	8.7019	0.0006
	Cxcl13	chemokine (C-X-C motif) ligand 13	spleen - mammary gland	4.3369	0.0047
	Cyp11b1	cytochrome P450 family 11 subfamily B member 1	adrenal	5.4150	0.0001
	Cyp1a2	cytochrome P450. family 1. subfamily a. polypeptide 2	liver	7.4701	0.0312
	Defa22	defensin. alpha. 22	intestine	6.3110	0.0102
	Dlk1	delta like non-canonical Notch ligand 1	limb - liver	4.0610	0.0080
	Dntt	deoxynucleotidyltransferase. terminal	thymus - bone marrow	5.5567	0.0003
	Dynlrb2	dynein light chain roadblock-type 2	testis- lung	2.6935	0.0139
	Ermn	ermin	brain	6.1440	0.0001
	Fabp6	fatty acid binding protein 6. ileal	intestine	7.5368	0.0014
	Fut7	fucosyltransferase 7	mammary gland	5.2695	0.0264
	Gcm1	glial cells missing homolog 1	placenta - kidney	6.0454	0.0003
	Gm6289 (Clca4c-ps)	chloride channel accessory 4C. pseudogene	intestine	10.2502	0.0004
	Gpx5	glutathione peroxidase 5	genital fat pad adult	4.7482	0.0010
	Gpx6	glutathione peroxidase 6	testis - genital fat pad adult	2.9562	0.0281
	Gstm7	glutathione S-transferase. mu 7	liver	3.1482	0.0480
	Gys2	glycogen synthase 2	liver	11.6204	0.0207
	Gzmb	granzyme B	intestine	5.1285	0.0450
	Hsd3b1	hydroxy-delta-5-steroid dehydrogenase. 3 beta- and steroid delta-isomerase 1	adrenal - ovary	4.0601	0.0001
	Igj (jchain)	immunoglobulin joining chain	intestine	2.2039	0.0003
	Ins2	insulin 2	duodenum - colon	5.2671	0.0367
	Insc	INSC spindle orientation adaptor protein	liver	6.9385	0.0332
	Itih3	inter-alpha trypsin inhibitor. heavy chain 3	liver	6.6714	0.0347
	Kcnmb1	potassium large conductance calcium-activated channel. subfamily M. beta membe	bladder - ovary	3.5618	0.0335
	Klk1b9	kallikrein 1-related peptidase b9	testis-kidney	4.8768	0.0115
	Krt12	keratin 12	brain	9.1004	0.0174
	Krt4	keratin 4	lung - stomach	2.5004	0.0404
	Lgals7	lectin. galactose binding. soluble 7	stomach-lung	2.5533	0.0101
	Mbl2	mannose-binding lectin (protein C) 2	liver	34.9644	0.0235
	Mup5	major urinary protein 5	liver	7.7335	0.0004
	Myl3	myosin. light polypeptide 3	heart	12.4529	0.0235
	Myoc	myocilin	bladder- brain	5.4003	0.0001
	Nts	neurotensin	intestine	4.5164	0.0040
	Padi6	peptidyl arginine deiminase. type VI	ovary	10.0716	0.0001
	Pgc	progastricsin	duodenum	12.2141	0.0005
	Piwil1	piwi-like RNA-mediated gene silencing 1	testis	5.1661	0.0001
	Pla2g12b	phospholipase A2. group XIIB	placenta - intestine	8.5702	0.0002
	Pla2g1b	phospholipase A2. group IB	stomach	6.7422	0.0340
	Pomc	pro-opiomelanocortin-alpha	testis - CNS	13.8359	0.0004
	Prl3b1	prolactin family 3. subfamily b. member 1	placenta	13.3400	0.0005
	Psca	prostate stem cell antigen	stomach	2.6411	0.0305
	Pyy	peptide YY	colon - intestine	3.1392	0.0243
	Rarres1	retinoic acid receptor responder (tazarotene induced) 1	liver - genital fat pat	5.0124	0.0016
	Rbp7	retinol binding protein 7. cellular	fat pad - heart	21.9595	0.0232
	Scrg1	scrapie responsive gene 1	brain	13.5691	0.0001
	Serpinb9b	serine (or cysteine) peptidase inhibitor. clade B. member 9b	placenta	15.0183	0.0108
	Serpinb9g	serine (or cysteine) peptidase inhibitor. clade B. member 9g	placenta	5.0895	0.0003
	Sftpc	surfactant associated protein C	lung	8.7768	0.0383
	Slc22a14	solute carrier family 22 (organic cation transporter). member 14	testis	4.2372	0.0006
	Slc38a5	solute carrier family 38. member 5	fat pad - ovary	9.0765	0.0188
	Sprr2i	small proline-rich protein 2I	stomach	7.0334	0.0397
	Tac2	tachykinin 2	liver	5.4785	0.0103
	Th	tyrosine hydroxylase	adrenal - brain	5.3596	0.0459
	Ugt2a3	UDP glucuronosyltransferase 2 family. polypeptide A3	liver - duodenum	6.8583	0.0006
	Vtcn1	V-set domain containing T cell activation inhibitor 1	fat pad - mammary gland	4.1635	0.0346
	Zmynd10	zinc finger. MYND domain containing 10	testis - kidney	2.4660	0.0397
**Fezf2-dependent TRAs**	Apol7c	apolipoprotein L 7c	intestine - spleen	10.0120	0.0104
	Calb1	calbindin 1	cerebellum - kidney	3.2992	0.0439
	Coch	cochlin	spleen - ovary	3.8648	0.0150
	Fabp7	fatty acid binding protein 7	brain	5.7656	0.0465
	H2-Ob	histocompatibility 2. O region beta locus	spleen - mammary gland	11.3795	0.0001
	Smtnl1	smoothelin-like 1	testis - mammary gland	3.4961	0.0163
	Sult1c2	sulfotransferase family. cytosolic. 1C. member 2	kidney - stomach	3.4840	0.0061
**Aire/Fezf2-dependent TRAs**	Apoa1	apolipoprotein A-I	liver - duodenum	2.1950	0.0305
	Apoa4	apolipoprotein A-IV	duodenum - intestine	2.0612	0.0057
	Apoc2	apolipoprotein C - II	duodenum - intestine	3.3456	0.0204
	Calb2	calbindin 2	brain	14.6915	0.0091
	Fabp9	fatty acid binding protein 9	testis	2.4346	0.0434
	Gstm3	glutathione S-transferase. mu 3	duodenum - intestine	14.4119	0.0064
	Pah	phenylalanine hydroxylase	kidney - liver	10.6327	0.0103
	Reg1	regenerating islet-derived 1	duodenum - intestine	7.2557	0.0005
	Saa2	serum amyloid A 2	colon - intestine	6.1821	0.0290
**Aire/Fezf2-independent TRAs**	Abcb4	ATP-binding cassette. sub-family B (MDR/TAP). member 4	liver	7.0771	0.0186
	Acacb	acetyl-Coenzyme A carboxylase beta	heart - mammary gland	2.5406	0.0004
	Adam12	a disintegrin and metallopeptidase domain 12	fat pad - mammary gland	2.6822	0.0455
	Adam33	a disintegrin and metallopeptidase domain 33	bladder - limb	3.1551	0.0338
	Adcyap1	adenylate cyclase activating polypeptide 1	CNS	12.1923	0.0137
	Aknad1	AKNA domain containing 1	testis	7.0766	0.0318
	Apoc1	apolipoprotein C-I	liver	2.1978	0.0024
	Atp4b	ATPase. H+/K+ exchanging. beta polypeptide	stomach	8.1920	0.0284
	Aurkb	aurora kinase B	thymus - liver	2.4643	0.0172
	Cadps	Ca2+-dependent secretion activator	CNS	2.3406	0.0001
	Calca	calcitonin/calcitonin-related polypeptide. alpha	thymus - intestine	4.0207	0.0016
	Calcb	calcitonin/calcitonin-related polypeptide. beta	thymus - intestine	4.2956	0.0001
	Ccl2	chemokine (C-C motif) ligand 2	stomach	3.1370	0.0300
	Ccl5	chemokine (C-C motif) ligand 5	intestine - mammary gland	2.3292	0.0188
	Cd3d	CD3 antigen. delta polypeptide	spleen	7.3832	0.0324
	Cd3e	CD3 antigen. epsilon polypeptide	thymus - spleen	4.8813	0.0029
	Cd8a	CD8 antigen. alpha chain	thymus - spleen	2.9968	0.0400
	Cd8b1	CD8 antigen. beta chain 1	thymus - spleen	3.6659	0.0442
	Celsr3	cadherin. EGF LAG seven-pass G-type receptor 3	brain	2.8167	0.0392
	Chek1	checkpoint kinase 1	CNS - liver	4.2620	0.0077
	Clec11a	C-type lectin domain family 11. member a	ovary-limb	2.2969	0.0258
	Crhbp	corticotropin releasing hormone binding protein	brain	2.1865	0.0339
	ooep	oocyte expressed protein	bladder - ovary	3.1144	0.0019
	Crybb1	crystallin. beta B1	genital fat pad - ovary	6.2468	0.0489
	Csn2	casein beta	intestine - mammary gland	3.8854	0.0001
	Ctla4	cytotoxic T-lymphocyte-associated protein 4	thymus - spleen	6.6578	0.0192
	Cux2	cut-like homeobox 2	brain	5.5350	0.0497

	Dhh	desert hedgehog	ovary - testis	4.3975	0.0234
	Dleu7	deleted in lymphocytic leukemia. 7	kidney - CNS	2.7752	0.0026
	Dusp15	dual specificity phosphatase-like 15	testis - cerebellum	5.6705	0.0162
	E2f2	E2F transcription factor 2	thymus - liver	2.2243	0.0246
	E2f7	E2F transcription factor 7	CNS - limb	2.8800	0.0348
	Eda	ectodysplasin-A	limb - fat pad	3.0075	0.0159
	Efcab9	EF-hand calcium binding domain 9	testis - spleen	5.2622	0.0331
	Efhc1	EF-hand domain (C-terminal) containing 1	testis - ovary	2.3821	0.0157
	Fam26f	family with sequence similarity 26. member F	spleen - mammary gland	3.4818	0.0344
	Fgf21	fibroblast growth factor 21	thymus - testis	2.6972	0.0001
	Flt3	FMS-like tyrosine kinase 3	cerebellum - spleen	4.3569	0.0144
	Frem2	Fras1 related extracellular matrix protein 2	limb - bladder	2.7537	0.0433
	Fxyd2	FXYD domain-containing ion transport regulator 2	kidney	2.0758	0.0049
	Gnmt	glycine N-methyltransferase	liver	3.6679	0.0337
	Grin2c	glutamate receptor. ionotropic. NMDA2C (epsilon 3)	cerebellum - brain	2.5578	0.0038
	H2-DMa	histocompatibility 2. class II. locus DMa	spleen - intestine	2.7030	0.0001
	H2-DMb1	histocompatibility 2. class II. locus Mb1	spleen - intestine	2.4298	0.0001
	H2-DMb2	histocompatibility 2. class II. locus Mb2	spleen - mammary gland	8.5206	0.0001
	H2-Eb2	histocompatibility 2. class II antigen E beta2	spleen - adult	5.0386	0.0002
	H2-Oa	histocompatibility 2. O region alpha locus	spleen - thymus	6.5328	0.0001
	Hamp	hepcidin antimicrobial peptide	liver	2.9829	0.0265
	Htra4	HtrA serine peptidase 4	adrenal - lung	3.4506	0.0123
	Hvcn1	hydrogen voltage-gated channel 1	spleen - mammary gland	2.6758	0.0247
	Il10ra	interleukin 10 receptor. alpha	thymus - spleen	2.8126	0.0416
	Il12a	interleukin 12a	spleen - lung	2.8994	0.0332
	Il22	interleukin 22	cerebellum - intestin	13.1907	0.0410
	Iqgap3	IQ motif containing GTPase activating protein 3	limb - CNS	2.8562	0.0115
	Itih2	inter-alpha trypsin inhibitor. heavy chain 2	liver	4.9636	0.0123
	Kif11	kinesin family member 11	CNS - liver	3.0915	0.0042
	Kif24	kinesin family member 24	testis - CNS	4.3120	0.0292
	Krt25	keratin 25	cerebellum - placenta	8.1643	0.0335
	Lck	lymphocyte protein tyrosine kinase	thymus - spleen	2.8682	0.0005
	Lrrc23	leucine rich repeat containing 23	lung - testis	2.3185	0.0064
	Lyve1	lymphatic vessel endothelial hyaluronan receptor 1	lung - liver	5.7736	0.0426
	Madcam1	mucosal vascular addressin cell adhesion molecule 1	spleen - intestine	8.9650	0.0009
	Mlf1	myeloid leukemia factor 1	testis - heart	2.0992	0.0310
	Mns1	meiosis-specific nuclear structural protein 1	liver	4.2458	0.0173
	Mylk2	myosin. light polypeptide kinase 2. skeletal muscle	mammary gland	7.1827	0.0279
	Mylk3	myosin. light polypeptide kinase 3. skeletal muscle	heart - liver	8.0759	0.0301
	Nppc	natriuretic peptide type C	ovary - cerebellum	3.2419	0.0170
	Nsg2	neuron specific gene family member 2	CNS - brain	4.5395	0.0217
	Nyx	nyctalopin	ovary - testis	6.3240	0.0364
	Ooep	oocyte expressed protein	bladder - ovary	3.1144	0.0019
	P2ry10	purinergic receptor P2Y. G-protein coupled 10	thymus - spleen	4.3968	0.0165
	Pdyn	prodynorphin	CNS - brain	7.0576	0.0229
	Pecam1	platelet/endothelial cell adhesion molecule 1	lung - fat pad	2.4883	0.0273
	Pglyrp2	peptidoglycan recognition protein 2	liver - thymus	2.5321	0.0021
	Pik3r6	phosphoinositide-3-kinase regulatory subunit 5	lung - mammary gland	5.8536	0.0386
	Plb1	phospholipase B1	intestine - testis	2.1379	0.0002
	Prg2	proteoglycan 2	liver	2.8337	0.0190
	Prg3	proteoglycan 3	liver	3.9672	0.0250
	Prph	peripherin	CNS	6.1944	0.0057
	Qrfpr	pyroglutamylated RFamide peptide receptor	brain	5.5623	0.0089
	Rag1	recombination activating gene 1	thymus - bone marrow	6.2759	0.0088
	Runx3	runt related transcription factor 3	spleen - thymus	2.9391	0.0034
	Ryr3	ryanodine receptor 3	CNS - bladder	2.7214	0.0279
	S100a14	S100 calcium binding protein A14	bladder - colon	2.2439	0.0001
	Scube2	signal peptide. CUB domain. EGF-like 2	lung - bladder	6.4017	0.0001
	Serpina12	serine (or cysteine) peptidase inhibitor. clade A	liver	4.4931	0.0139
	Serpinb12	serine (or cysteine) peptidase inhibitor. member b	lung - stomach	2.1352	0.0136
	Slc17a3	solute carrier family 17 member 3	kidney - liver	6.4444	0.0001
	Slc2a6	solute carrier family 2 (facilitated glucose transporter). member 6	cerebellum - spleen	6.6805	0.0054
	Slc7a15	solute carrier family 7 (cationic amino acid transporter. y+ system). member 15	intestine - duodenum	6.5729	0.0001
	Sncg	synuclein. gamma	fat pad	2.4389	0.0013
	Snx29	sorting nexin 29	kidney - spleen	3.3393	0.0004
	Sostdc1	sclerostin domain containing 1	kidney - limb	2.3161	0.0030
	Spag16	sperm associated antigen 16	testis	3.7948	0.0370
	Spic	Spi-C transcription factor	liver - spleen	5.9688	0.0441
	Spon2	spondin 2. extracellular matrix protein	colon	2.5576	0.0031
	Stmn2	stathmin-like 2	CNS - brain	2.5760	0.0014
	Stra6	stimulated by retinoic acid gene 6	ovary	3.4318	0.0007
	Tbx21	T-box 21	spleen - brain	2.2845	0.0359
	Tcf7	transcription factor 7. T cell specific	thymus - spleen	3.5848	0.0001
	Tmem63c	transmembrane protein 63c	cerebellum - testis	5.4051	0.0450
	Trib3	tribbles pseudokinase 3	duodenum - intestine	3.2222	0.0209
	Tspyl5	testis-specific protein. Y-encoded-like 5	brain - testis	2.4381	0.0244
	Twist1	twist basic helix-loop-helix transcription factor 1	limb. CNS	5.8015	0.0482
	Ubash3a	ubiquitin associated and SH3 domain containing. A	thymus - liver	8.4886	0.0259
	Ubd	ubiquitin D	thymus - intestine	2.7031	0.0001
	Upk1a	uroplakin 1A	bladder - ovary	10.9666	0.0104
	Upk3b	uroplakin 1B	bladder - lung	2.4128	0.0452
